# Social media use for nutrition outcomes in young adults: a mixed-methods systematic review

**DOI:** 10.1186/s12966-018-0696-y

**Published:** 2018-07-24

**Authors:** Karen M. Klassen, Caitlin H. Douglass, Linda Brennan, Helen Truby, Megan S. C. Lim

**Affiliations:** 1 Department of Nutrition, Dietetics & Food, Monash University, Level 1 264 Ferntree Gully Road, Notting Hill, VIC 3168 Australia; 2 Burnet Institute, Melbourne, Australia; 3 School of Media and Communications, RMIT University, Melbourne, Australia; 4 Melbourne School of Population and Global Health, University of Melbourne, Melbourne, Australia

**Keywords:** Young adults, Social media, Healthy eating, Weight

## Abstract

**Background:**

Social media has been widely adopted by young adults, consequently health researchers are looking for ways to leverage this engagement with social media for the delivery of interventions and health promotion campaigns. Weight gain and sub-optimal dietary choices are common in young adults, and social media may be a potential tool to facilitate and support healthier choices.

**Methods:**

We conducted a mixed-methods systematic review of studies examining social media use for nutrition-related outcomes in young adults. Seven databases [EBscohost, ERIC, ProQuest Central, PubMed, Ovid, Scopus, and Emerald] were systematically searched; 1225 abstracts were screened, and 47 full-text articles were assessed for eligibility. Study designs included both quantitative, such as experimental and observational studies, and qualitative, such as focus groups and interviews, approaches. Quality was assessed using the Mixed Methods Appraisal Tool. Quantitative and qualitative results were examined separately, and then synthesized.

**Results:**

Twenty-one studies were included although their use of social media was highly variable. The main purpose of social media was to provide information and social support to participants. In the nine randomized controlled trials, social media was used as one aspect of a multi-faceted intervention. Interventions had a positive statistically significant impact on nutritional outcomes in 1/9 trials. Engagement with the social media component of interventions varied, from 3 to 69%. Young adults appear to be open to receiving healthy eating and recipe tips through social media, however, they are reluctant to share personal weight-related information on their online social networks.

**Conclusions:**

Information-dissemination is now an acceptable use of social media by young adults. Using social media effectively for social support, either via private groups or public pages, requires careful evaluation as its effectiveness is yet to be demonstrated in experimental designs. Concerns about public social media use may be a contributing factor to poor engagement with social media in research intervention studies aimed at influencing weight. Future research should consider how to best engage with young adults using social media, how to more effectively use social media to support young adults and to facilitate social and peer-to-peer support in making healthier choices.

**Electronic supplementary material:**

The online version of this article (10.1186/s12966-018-0696-y) contains supplementary material, which is available to authorized users.

## Background

Overweight and obesity is a major public health problem leading to 2.8 million deaths globally each year [1]. When examining the impact of dietary components within the context of global burden of disease, poor dietary patterns are responsible for more deaths than any other modifiable risk factor in non-communicable disease excluding smoking [2]. Obesity (high body mass index) and its associated co-morbidities such as hypertension and hyperglycemia feature as preventable conditions treatable by optimizing dietary patterns and increasing physical activity.

Young adulthood, commonly defined as aged 18–35 years, is a unique life stage as most individuals transition from a dependent life as an adolescent, living with family and going to school, to becoming independent and self-sufficient [3]. Increased autonomy around eating choices, developing cooking skills and finding physical activity that is not reliant on organized school sport are all important factors that can influence weight changes during this life stage [4, 5]. This life stage is characterized by a rapid increase in weight trajectory which makes young adulthood a window of opportunity where obesity prevention strategies may have a great impact [6, 7]. Surveillance of young adults dietary patterns show it can be characterized by low fruit and vegetable [8, 9] and high sugar-sweetened beverage consumption [10–12]. These specific diet choices, probably contribute to weight gain plus establish unhealthy eating patterns which track into later adulthood. The challenge is how to engage young adults in making and adopting a long term dietary pattern that will help prevent weight gain at a time when their health is not necessarily high in their priorities. Today, in addition to being influenced by peers, family and traditional media, young adults are continuously exposed to information via social media [13], which may influence social norms and their behavior [14]. Social media can be defined as any web-based communication channel dedicated to community-based input, interaction, content-sharing and/or collaboration i.e. used for online social networking [15]. This can include social network channels, such as well-known, publicly available platforms (e.g. Facebook, YouTube, Snapchat), or purpose-built, private discussion forums for ‘closed’ groups.

Health, nutrition and food are all common topics posted on social media by food and wellness bloggers [16], health organizations and regular users of social media. In addition to social media users organically posting about food, young adults are being bombarded by food, often junk food, messages sponsored by food industry organizations with a commercial interest [17].

Many health professionals recognize that social media provides an opportunity to reach and engage with young adults that may not otherwise seek out health professionals in more traditional settings [18]. Social media can act as a platform to deliver interventions and health promotion campaigns, increase exposure to evidence-based health messages and encourage young adults to participate and engage with interventions. Although social media is used almost ubiquitously by young adults [13, 19], it is unknown if they are engaging, or wish to use it to engage with, health promotion content. Celebrities, the food industry and “lifestyle gurus” compete for attention on social media platforms and it is unclear if young adults will accept social media to engage with health-related interventions led by health professionals [20] who may appear boring and unattractive in this contested space. The art of communicating health messages using social media in the context of weight loss is an emerging area of research requiring nutritionists to work with communications, media and marketing professionals to understand how to engage and interact with young adults to change diet and activity patterns [21]. Lim et al. [22], has reviewed how to use social media with regards to alcohol intake, however there has been no similar systematic review that focuses on nutrition-related outcomes, such as dietary intake.

Previous systematic reviews have examined social media use for health-related behaviors [23–27]; these have varied in the definition of social media itself, whether referring to online social networking specifically or including a broader definition of all social media channels or platforms included and how social media was used. Study design and outcomes have varied also within the papers included in each review, as this is a constantly evolving space. Some reviews included only RCTs [25, 26], while others included a wider variety of designs [18, 23]. Outcomes varied from nutrition, physical activity, smoking to other health-related outcomes. In one review, use of on-line social media was associated with only small improvements in outcomes [27]. In a review exploring the use of social media in adolescents and young adults [23], effectiveness was not evaluated, however, Yonker et al. found that social media was being widely used in research for a variety of purposes, such as recruitment, for observing participants, collecting data and providing health information. Only a small number of papers included in these previous reviews examined nutrition-related outcomes. In a review examining only weight-related outcomes, Willis et al. found that complex interventions specifically using online social networking, as a component of the intervention were not associated with improvements in weight management outcomes when compared with control groups [24]. These previous systematic reviews have focused solely on examining the effectiveness of social media for health outcomes; however, they do not adequately describe how and why social media was or was not effective. Vandelanotte and Maher [28] argue that randomized controlled trials (RCTs) should be used to evaluate efficacy of online social networking as a tool, but that ecological, pragmatic studies should be used to evaluate the effectiveness and reach of social media. It is also important to evaluate social media research taking place ‘organically’ and learn from how people are actually using the popular, publicly available social media platforms to share, learn and engage with their nutritional health (specifically healthy eating/diet/food).

Our previous systematic review of evaluation practices for social media interventions also described the benefits of using study designs other than RCTs to improve the evaluation of interventions taking place in social media beyond narrow and tightly controlled trials [29]. Further, it recommended evaluating the reach and engagement of an intervention, as well as effectiveness, and the use of mixed-methods evaluation when possible [29]. This present review deliberately adopts a mixed methods approach in order to expand our understanding of how young adults want to use social media to learn about nutrition in addition to synthesizing the results from experimental research about nutrition outcomes, and understand how and why interventions worked, or did not work. Furthermore, it is important to focus on young adults as a distinct group, as they have different uses of social media [13], different health engagement and are less likely to be overweight already compared with older adults [30].

The aims of this review were to:

1. Describe how young adults use social media in nutrition-related interventions.
2. Evaluate engagement metrics used in social media interventions for nutrition-related outcomes in young adults.
3. Understand whether engagement with social media in nutrition-related interventions improves nutrition-related outcomes.
4. Explore the functions of social media and how they these can be leveraged for greatest impact in nutrition-related interventions.
5. Understand how young adults use social media for nutrition-related behaviors.

## Methods

This systematic review was conducted using a mixed-methods approach according to the procedures outlined in the Joanna Briggs Institute’s Methodology for JBI Mixed Methods Reviews [31]. This method uses a segregated approach to synthesis. Quantitative and qualitative studies are analyzed separately prior to finally synthesizing the two analyses together.

### Inclusion and exclusion criteria

#### Types of studies

All types of empirical study designs (quantitative and qualitative) were included in order to achieve the multi-faceted research aims. Examples of quantitative studies could include: randomized controlled trials, experimental and quasi-experimental designs, observational cohort studies or cross-sectional studies. Examples of qualitative studies could include those studies using focus groups or interviews. Published and unpublished [32] studies were included in recognition that social media is a rapidly evolving platform. Study quality has been reported on all included studies. Guidelines, protocols, opinion pieces, conference abstracts and review articles were excluded.

#### Types of participants

Participants were healthy and/or overweight or obese young adults and were not pregnant. Young adults are typically defined as aged between 18 and 35 years, but we acknowledge there is no consistently used age range, so if a study reported focusing on young adults, it was considered for inclusion in this review.

#### Types of intervention(s)/phenomena of interest

Social media was defined as ‘any web-based communications channel dedicated to community-based input, interaction, content-sharing and/or collaboration’. This definition includes both the popular publicly available platforms (e.g. Facebook, YouTube, Snapchat), as well as any website and applications dedicated to forums (e.g. www.reddit.com), micro-blogging (e.g. www.twitter.com), social networking (e.g. www.facebook.com), social bookmarking (e.g. https://pinboard.in/), social curation (e.g. https://en.wikipedia.org/wiki/Main_Page or using Twitter to form lists) and wikis (e.g. en.wikipedia.org) [15].

Studies were included if they used social media as a component of an intervention or explored social media as an exposure or phenomena of interest.

#### Types of outcomes

Outcome measures relating to nutrition-related outcomes were included: body composition (weight, body mass index (BMI), waist circumference or other body composition measures) or dietary intake (foods or nutrients). Publications evaluating only physical activity or alcohol outcomes, or antecedents of healthy eating behavior only were excluded.

### Literature search strategy

Seven databases [EBscohost (including: CINAHL Plus, MEDLINE, Communication & Mass Media Complete, Business source complete), ERIC (via Proquest), ProQuest Central, PubMed, Ovid (including: psycARTICLES full text, JBI, EBM reviews-acp, cochrane, hta, nhs, AMED, psycBOOKS, PsycINFO 1987–2017), Scopus, and Emerald] were searched using predetermined keywords (Keywords included any combination of a social media term AND young adult term AND nutrition term and NOT disease with additional detail found in Additional file 1: Table S1), limited to studies published in English from 2000 until 20 April 2017 (this corresponds to the emergence of social media use by the mainstream population [33], and the beginning of its use in health research [34, 35]).

### Data management

Results from each database were imported into Endnote for an initial duplicate removal process. This file was then imported into Covidence software (Covidence systematic review software, Veritas Health Innovation, Melbourne, Australia. Available at https://www.covidence.org/home) where the remaining steps occurred.

### Data screening / study selection

Two reviewers (KK and CB) independently screened the articles by title and abstract to determine potential eligibility. For articles requiring further investigation, two reviewers (KK and CD) independently read the full text of the articles before deciding the final list of articles for inclusion in the review. Disagreement between the reviewers was resolved by discussion or consultation with a third reviewer (ML). Authors who published articles, which included participants in the inclusion age bracket, but did not include reported outcomes for young adults were emailed and age-specific results were requested [36, 37].

### Quality assessment

Quantitative, qualitative and mixed-methods papers were assessed by two independent reviewers (KK and CD) for methodological validity using the Mixed Methods Appraisal Tool (MMAT), a standardized critical appraisal instrument [38]. Inter-rater reliability was calculated by assigning “1” to each question with agreement between the two reviewers and “0” to each question with difference between the two reviewers. A consensus was achieved for all questions through discussion. The MMAT includes questions for various study designs, and each subset includes four to six questions (see Additional file 1: Table S3). An overall score for quality was calculated by counting the number of “low risk” scores for each category. If the study scored > 4, it was considered “high quality”, 3–4 was “moderate quality” and < 3 was “low quality”.

### Data extraction

Quantitative data extracted included details about the interventions, populations, study methods, results and outcomes of significance to the review question and specific objectives. Qualitative data extracted from papers included details about the populations, study methods, phenomena of interest description and findings of significance to the review questions and specific objectives.

### Data synthesis

Social media use as part of an intervention or an exposure or phenomenon of interest was categorized based on previously identified categories and commonly designated categories of social media use (see Additional file 1: Table S2).

Level of engagement with interventions was evaluated using quantitative data and is presented descriptively. Measures of engagement included ‘likes’ and comments on study posts about recipes/nutrition, number of participant posts, responses to events and page views.

The included RCTs investigated the effectiveness of complex interventions, which included social media. As there were substantial differences and heterogeneity between the interventions, it was not possible to conduct a meta-analysis. The findings evaluating whether social media engagement assisted participants in improving their nutrition-related outcomes are presented in narrative form.

Qualitative research findings were, where possible pooled using the JBI-QARI tool [31]. This involved reading through the findings from all studies and categorizing these findings based on similarity of meaning. These categories were then synthesized to generate a set of themes that represented that synthesis. Finally, quantitative and qualitative findings were synthesized by discovering where the qualitative themes could explain the ‘why’ and ‘how’ of the quantitative findings.

## Results

**Fig. 1:**
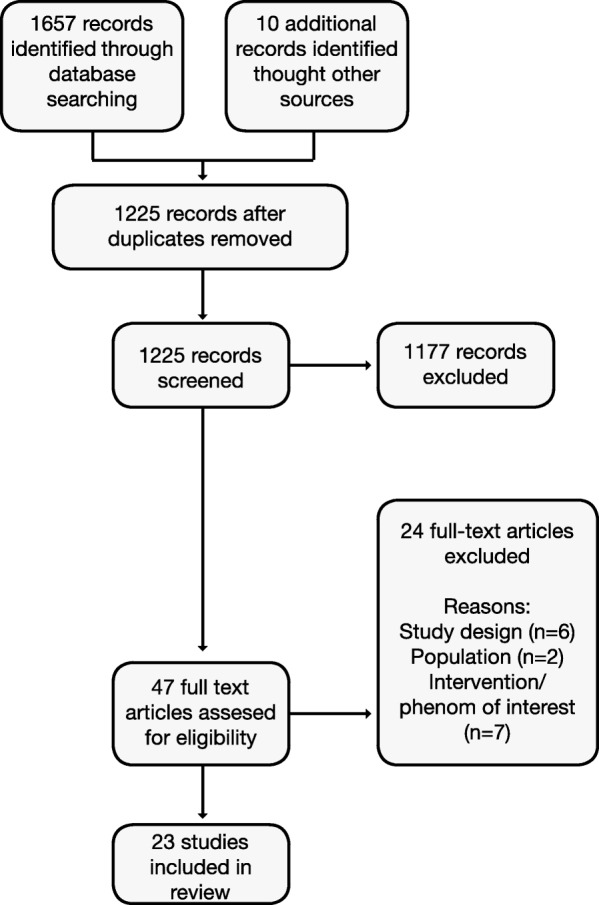
Flow diagram of study selection

Figure 1 shows the flow of studies through the selection process. In total, 23 studies were included (Tables 1 and 2). Studies included ten (48%) RCTs (however, two papers reported different outcomes for the same intervention, therefore only nine interventions were included), two (10%) process evaluations following RCTs that included mixed methods, one process evaluation that included quantitative survey results only, four (19%) quasi-experimental, one (5%) mixed-methods and three (14%) qualitative studies. The majority of studies took place in the United States, within the past 5 years, with the majority of participants being female with a mean age of < 25 years and 17/23 (74%) recruited university or college students. Over half (14/23) had a mean BMI ≥25, reflecting the weight loss aim of many studies. BMI and/or weight were outcomes reported in the majority of RCTs. Dietary intake was reported using different measures, often related to fruit and/or vegetable intake.

**Table 1:** Description of all included studies about social media and nutrition-related outcomes in young adults

Source	Methods	Population				Study characteristics			Quality appraisal
Author, year, country	Method/study design	n	Mean age	Mean BMI or % overweight and/or obese	% female	Description of social media examined (as a component of an intervention or phenomenon of interest)^a^	Primary outcome(s) (If relevant)	Length of follow-up if relevant (months)	Quality score
Gow, 2010 [43], United States	RCT: 4 arms	159	18.1	24.4 (5.1)	74%	A private discussion board with scheduled weekly group discussions and asynchronous discussion groups facilitated by researcher.	BMI, weight, Fruit and vegetable (combined) intake (serves/day)	4	Poor
Napolitano, 2013 [39], United States	RCT: 3 arms	52	20.5 (2.2)	31.4 (5.3)	87%	A private Facebook group containing posts with content such as handouts and podcasts, suggested caloric intake, access to polls and healthy activity or eating event invitations. Used group postings and messages.	Weight	2	High
Ashton, 2017 [6], Australia	RCT	50	22.1 (2.0)	25.5 (4.6)	0%	A private Facebook discussion group to facilitate social support, send reminders for upcoming face-to- face sessions and send notifications for new material added to the website	BMI, weight, Fruit & Vegetable intake (serves/day), Diet Quality, Energy Intake (kJ/day), body fat mass (g)	3	High
Beetham, 2015 [40], United States	RCT	46	18.6	100%	100%	A private Facebook group containing only members of group (*n* = 7–9); expected to post once weekly as well as the lifestyle coaches from their groups. The facilitators shared tips about nutrition, physical activity, inspirational quotes, reminders to read weekly material and attend sessions, and self-compassion and prompted feedback about the weekly lesson.	BMI, weight	6	Poor
Dadkhah [51], 2013 [49], United States	RCT	216	18 (NR for overall)	22 (NR for overall)	65%	A Facebook group, similar to Phase 2, with weekly posts of relevant tools, news articles, educational Web sites, pages, images, or short texts that supported daily tips.	BMI, weight, energy intake (kcal/day)	7	Poor
Godino, 2016 [42], United States	RCT	404	22.7 (3.8)	100%	70%	A public Facebook group allowing for social support, accountability, and healthy social norms from existing social networks. The research team delivered 17 challenges and campaigns that were often culturally themed and promoted changes to weight-related behaviours.	BMI, weight	24	High
Hebden, 2014 [49], Australia	RCT	51	23 (NR for overall)	mean BMI 27 (NR for overall)	80%	A private Internet forum where participants and investigator could to contribute comments, questions and information. New healthy eating information was posted by the investigator biweekly.	BMI, weight, Vegetable intake, fruit intake, sugar-sweetened beverages, energy-dense takeaway meals	3	High
Laska 2016 [47], United States	RCT	441	22.7 (5.0)	25.4 (3.8)	68%	see Laska 2016	Fast food, sugary beverages, eat breakfast 5–7 days/week; weekly meals prepared at home	24	High
Lytle, 2017 [63], United States	RCT	441	22.7 (5.0)	25.4 (3.8)	68%	see Laska 2016	BMI, weight, waist circumference, % body fat	24	High
Partridge, 2015 [64], Australia	RCT	250	27.7 (4.9)	27.1 (2.5)	61%	A community blog (no more information given).	BMI, weight	3	Moderate
Dadkhah, 2013 [51], United States	Qualitative: focus groups	25	18.0 (0.5)	NR	40%	Formative research: to determine the need for a weight gain prevention program using social media for first-year college students	n/a	n/a	Moderate
Dennison, 2013 [52], United Kingdom	Qualitative: focus groups	19	23.8 (7.9)	NR	68%	Exploring young adult perspectives on apps relating to health behaviour change.	n/a	n/a	High
Vaterlaus, 2015 [65], United States	Qualitative: focus groups and interviews	32	20.4	26%	79%	Exploring young adult perceptions of social media on health behaviours.	n/a	n/a	High
Laska, 2016 [47], United States	Process evaluation (of Lytle 2015). Quantitative methods; survey data and engagement metrics.	224	22.9 (5.0)	25.4 (3.8)	67%	A private social networking and support website with a discussion forum available to participants and a limited number of their invited guests was designed to reinforce, inform and encourage exchange and support between participants. Students were encouraged to track their weight and up to 10 weight-related behaviours on the website. Trained interventionists interacted with participants through the website and there was an “Ask the Expert” section where students could ask confidential questions about a personal challenge or health issue. The website included articles, recipes, quizzes, videos and ways to accumulate points for prizes.	n/a	n/a	Poor
Merchant, 2014 [46], United States	Process evaluation (of Godino 2016). Mixed methods: engagement analytics and semi-structured interviews.	199	22.0 (3.8)	28.7 (3.5)	70%	Participants were invited to ‘like’ an open Facebook page. Non-study participants could also like the page, view content and interact with it. Behaviour change campaigns were posted, as well as healthy tips and tailored messages from the health coach. Participants were encouraged to self-monitor their diet and physical activity on Facebook.	n/a	n/a	Moderate
Partridge, 2016 [50], Australia	Process evaluation (or Partridge 2015). Mixed methods: survey data, engagement metrics and semi-structured interviews	110	NR	NR	NR	A community blog (no more information given).	n/a	n/a	Moderate
Pappa, 2017 [48], Brazil	Mixed methods; engagement analytics and content analysis.	754	26 (6)	NR	57%	Reddit is a public social media site. /r “LoseIt” is a subreddit community where people interact about weight loss issues.	n/a	51^a^	Poor
Chung, 2016 [41], United States	Experimental; single arm	12	20.3 (overweight group), 19.0 (healthy weight group)	58%	67%	A private Twitter group where participants received both text and photo-based Tweets from the study team that focused on health messages; encouragements and reminders to wear their Fitbits and log their dietary intake.Participants were encouraged to post questions to the study team or to their Twitter group. The study team posted questions to the group on topics such as “What small change are you going to make this week?” to encourage interactivity and to tailor message content to participant needs. Fitbit accounts were set up to autotweet daily steps and distance travelled to the assigned private Twitter group so that individuals could see how others were doing, which was the basis of some of the competitions.	n/a	2	Poor
Dadkhah 2013 [51], United States	Experimental; single arm	88	18.0 (0.4)	23	77%	A Facebook group (not identified as public or private) where daily health tips were shared. Participants chose the timing of the postings through Facebook.	BMI, weight, energy intake (kcal/day)	7	Moderate
Harvey-Berino, 2012 [44], United States	Experimental; single arm	336	NR	53%	87%	A bulletin board and discussion forum where 1-h weekly “group meetings” were led by an interventionist trained in behaviour modification and online facilitation. The bulletin board could be used for group communication.	BMI, weight	4	Poor
Meng, 2017 [45], United States	Experimental: 5 arms	111	19.9 (1.7)	22.7 (3.0)	67%	A private community group consisting of 3 modules: 1) group goal; 2) self-track message wall to post their F&V consumptions 3) bar graph illustrating weekly summaries. Groups consisted of 1 participant and “confederates” i.e. fake people.	Fruit and vegetable intake	1	Moderate

^a^Use predefined categories found in methods

**Table 2:** Quantitative study results

Author, Year	Country	Description of arms	Outcomes	Quantitative Results
Ashton, 2017	Australia	2 arms: intervention versus control (no treatment)	BMI, weight, Fruit & Vegetable intake (serves/day), Diet Quality, Energy Intake (kJ/day), body fat mass (g)	BMI: Mean difference change in BMI between intervention and control at 3 months: − 0.50 [− 0.89, − 0.11]
Beetham, 2015	United States	2 arms: Intervention #1 = Technology plus in-person; Intervention #2 = technology only No control arm group, therefore excluded from meta-analyses.	BMI, weight	BMI: Mean change of BMI at 8 weeks (intervention group #1): − 0.34 (s.d. 1.01); (intervention group #2): − 0.72 (s.d. 0.73).
Chung, 2017	United States	1 arm	Fruit and vegetable intake, SSB intake	Overweight group: increased fruit intake by mean 2.1 servings & vegetables by mean 2.5 serving & decreased SSB by 1.2 beverages; Healthy weight group: increased fruit intake by mean 1.8 servings & vegetables 0.5 servings & decreased SSB by 0.3 beverages. Measures of variance and statistical significance were not reported.
Dadkhah, 2013	United States	2 arms: Intervention versus control	BMI, weight, energy intake (kcal/day)	BMI: Mean difference between intervention and control at 7 months: 0.75 [0.62, 0.88]
Gow, 2010	United States	4 arms: Intervention #1: Internet only; #2: Feedback only; #3: combined Internet and feedback; #4: control	BMI, weight, Fruit and vegetable (combined) intake (serves/day)	BMI: Mean difference between intervention and control at 6 weeks: 0.91 [−1.23, 3.05]
Harvey-Berino, 2012	United States	1 arm; analysis was presented from 4 groups: Overweight versus healthy weight; and weight loss goal versus healthy lifestyle goal	BMI, weight	Results were reported for 4 groups. BMI: Mean difference between 4 months and baseline in overweight group with goal of weight loss: −1.0 [−2.47, 0.47]
Hebden, 2014	Australia	2 arms: Intervention versus control (received paper booklet with diet and physical activity information and one appointment with a dietitian)	BMI, weight, Vegetable intake, fruit intake, sugar-sweetened beverages, energy-dense takeaway meals	BMI: Mean difference between intervention and control at 3 months, adjusted for baseline data: −0.11 [−0.66, 0.43]; Vegetable, fruit, SSB: no statistical differences between interventionand control arms at 3 months
Laska & Lytle, 2017	United States	2 arms: Intervention versus control (received health promotion information quarterly)	BMI, weight, waist circumference, % body fat	BMI: Mean difference between intervention and control at 24 months: −0.20 [− 0.98, 0.58]
Laska, 2016	United States	see Laska and Lytle	Fast food, sugary beverages, eat breakfast 5–7 days/week; weekly meals prepared at home	SSB: Mean difference between intervention and control at 24 months: −0.10 [− 0.38, 0.18]
Meng, 2017	United States	5 arms: # 1: demographically similar, incremental change; # 2 demographically diverse/incremental changes; # 3 demographically similar/ ideal changes; # 4 demographically diverse/ideal changes; and #5:control	Fruit and vegetable intake	Not reported by pre-assigned groups
Merchant, 2014 & Godino 2016	United States	2 arms: intervention versus control (access o website with general health information and quarterly newsletters)	BMI, weight	BMI: Mean difference between intervention and control at 24 months: −0.30 [− 0.89, 0.29]
Napolitano, 2013	United States	3 arms: Intervention #1: Facebook only; Intervention #2: Facebook PLUS goal setting/ self-monitoring/social support	Weight	Weight: Mean difference change of weight between intervention and control at 8 weeks: −0.39 [−2.07, 1.29]
Pappa, 2017	Brazil	All members of r/Loseit forum; active users with weight data were included in quantitative analysis	Weight	3.7%(28/754) of users gained weight (mean 3.88%, SD 4.04), 3.5%(25/754) maintained weight, and 92.9% (701/754) lost weight.
Partridge, 2015 & Partridge, 2016	Australia	2 arms: Intervention versus control	BMI, weight	BMI: Mean difference between intervention and control at 3 months: −0.40 [−0.91, 0.11]

### Quality assessment

Study quality was assessed using the MMAT [38] for all studies. Inter-rater reliability for the initial assessment of studies was 90% (103 questions agreed/115 questions). Eight studies (38%) had a high overall quality score, seven had a medium score and eight were rated as poor quality (see Table 1).

In RCTs, allocation concealment was most poorly reported, followed by sequence generation and high attrition. Attrition varied widely between 4% [39] to 72% [40]. Only four out of 10 studies reporting RCT outcomes employed an intention-to-treat analysis. In qualitative studies, some did not give appropriate consideration to how findings relate to researchers’ influence, nor to how findings relate to the context. In mixed methods and/or process evaluations, there was little integration of qualitative and quantitative data or results relevant to address the research question objective. In the quasi-experimental studies, recruitment methods introduced selection bias, and some measurement tools were not validated (see Additional file 1: Table S3).

### Aim 1: Describe how young adults use social media in nutrition-related interventions

**Fig. 2:**
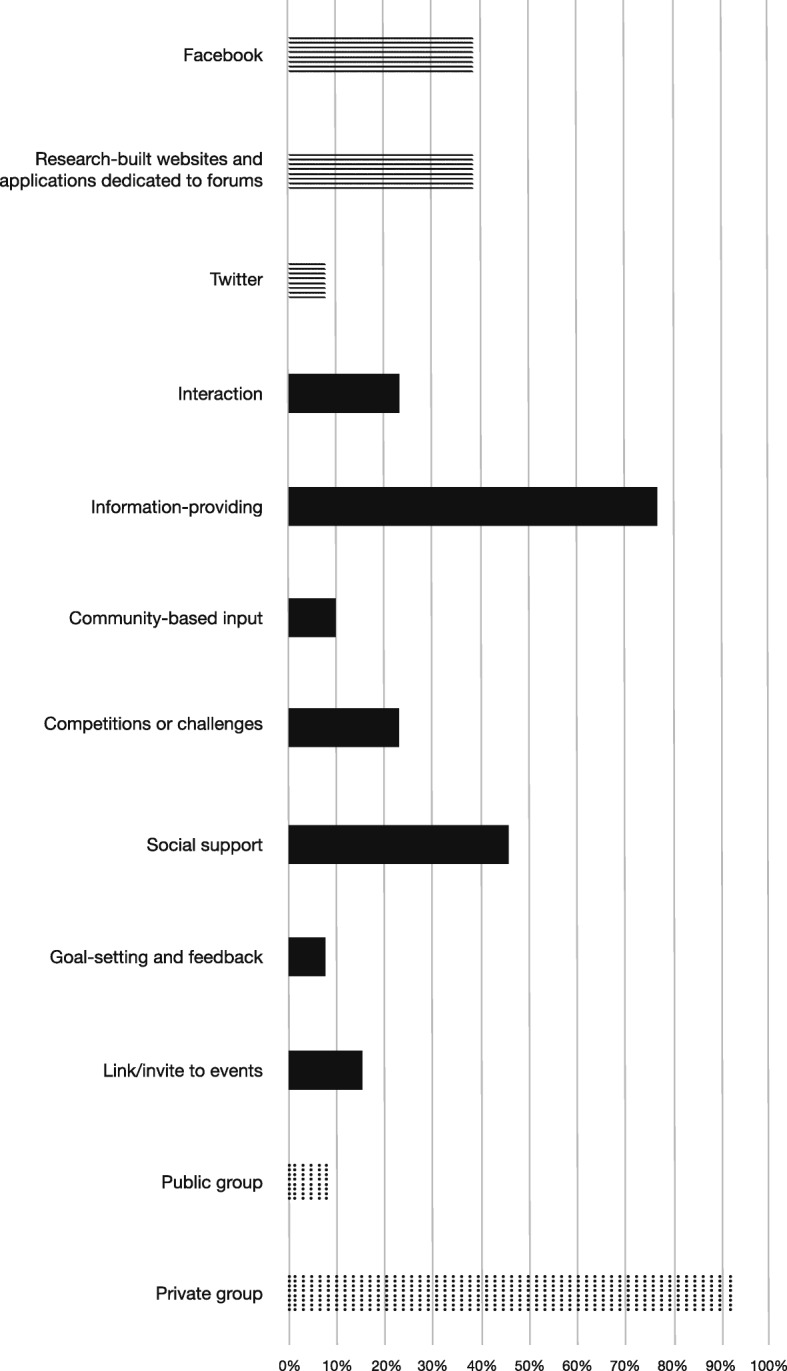
Characteristics of social media used as a part of interventions in 13 studies. *The wavy lines pattern indicates the type of platform used. Black indicates the purpose of social media. Dots indicate the privacy settings of the social media platform

Many of the experimental studies included complex interventions where social media was only one component of a complex intervention. Other components included websites with a resource library, behavior tracking devices or tools, personalized food and nutrition reports, short message service (SMS) reminders, group sessions, coaching, smartphone applications and others (Table 1). For many studies, social media was central to the delivery and efficacy of the intervention, for example when it was used to deliver all intervention content [41], and post interactive content and events [42]. In other studies, it was a minor component, for example, as an infrequently-used discussion board [43]. The mean number of components in the 13 included interventions was 4.2 (range 2–7). There were a variety of purposes and functions of social media (Fig. 2). The majority of the experimental studies used private or closed social networking groups on various social media platforms, mainly Facebook or purpose-built online forums. The main purpose of social media stated by the authors was to provide information, followed by providing social support to participants. Two of the three experimental (non-RCT) studies used public social media channels to deliver the core of their intervention.

### Aim 2: Evaluate engagement metrics used in social media interventions for nutrition-related outcomes in young adults

Nine studies examined engagement, including these measures: ‘likes’ and comments on study posts about recipes/nutrition, number of participant posts, responses to events and page views. Gow et al. [43], Harvey-Berino et al. [44] and Meng et al. [45] did not evaluate engagement or acceptability of the social media component in their interventions.

Engagement varied widely from 3 to 69% of participants engaging with social media in those studies reporting overall engagement with the social media component. Chung et al. [41] reported that their 12 participants tweeted 310 times over 2 months, with more in the overweight/obese group tweeting compared with the healthy weight group. Merchant et al.’s [46] interim analysis of the Godino et al. [42] RCT found a decline in the number of interactions on Facebook over time, however, investigators posted regular campaigns, which somewhat maintained engagement. Over 22 months, 137/199 (69%) engaged with their study Facebook page at least once. A small number of users (*n* = 32) interacted the most with their Facebook page (81% of all interactions). Laska et al. [47] also reported a declining engagement over their 24-month intervention; during the first month, more than half of participants logged in to the intervention website and logged their weight, which declined to 20–40% range for the rest of the intervention period. Although only 8/35 (23%) of participants in the two intervention arms using Facebook “liked” study-related posts in Napolitano et al. [39], 80% of participants responded via Facebook to events at least once, and 60% actively engaged with the Facebook content by making posts or comments on the Facebook page at least once during the short, 2-month intervention period. There were higher levels of engagement with the other major component of their intervention: SMS feedback.

In an analysis of actual members on an online weight loss group, Pappa et al. [48] found that 43% of users (107,886 of 252,279 users) posted at least once over the 4 years of analysis. Two percent of users contributed five times more posts than others. Hebden et al. [49] found that only two participants out of 26 in the intervention arm (8%) interacted with their Internet forum by posting information. Social media was the least-used component of the five intervention components. Partridge et al. [50] reported that only four out of 125 participants in the intervention arm (3%) logged on or used the community blog and this was the least-used component of the seven components of their intervention. Ashton et al. [6] found that in an evaluation of an online intervention where 25 young men used a private Facebook group, only one (out of 23 total posts) post was made by a participant. Beetham [40] reported that the mean use of the Facebook group was once (during the 2-month intervention) and that it was the least used component of the intervention. These four studies with low social media engagement all included at least five different components in their interventions.

### Aim 3: Understand whether engagement with social media in nutrition-related interventions improves nutrition-related outcomes

Fourteen studies examined effectiveness by measuring the effect of their interventions including social media on a nutrition-related outcome. Outcomes commonly assessed across studies were BMI, weight, energy intake, fruit and/or vegetable intake, and sugar-sweetened beverage intake (Table 1). As the interventions varied widely, with some only including social media as a platform for providing information, a meta-analysis of outcomes was not possible, as it could not accurately evaluate the effect of social media on nutrition-related outcomes.

Of ten included studies reporting outcomes from RCTs, seven included BMI and eight included weight as outcomes. The interventions containing a social media component did not have a positive effect on either outcome for eight of the nine studies when comparing the intervention with control groups. One study found that their pilot intervention led to a significant difference in BMI between the intervention and control arms at the end of 3 months [6]. Fruit, vegetable and SSB intakes did not differ between intervention and control groups for any studies evaluated. Chung et al. [41] did not report outcomes by assigned arm, but participants overall increased fruit and vegetable intake by 92% and decreased sugar-sweetened beverage intake by 67%. Dadkhahs’ single arm experimental study [51] reported a decrease in energy intake and Harvey-Berino et al. [44] reported that all groups decreased BMI. For those participants who had a goal to lose weight, overweight participants had the greatest weight loss (− 6.1 kg; 95% CI -3.3 to − 2.3).

### Aim 4: Explore the functions of social media and how they these can be leveraged for greatest impact in nutrition-related interventions

The functions of social media were described for aim 1 (Fig. 2). To understand how these functions can be leveraged for greatest impact and determine the acceptability of these functions, we report findings from formative research from intervention planning, and retrospective examination of interventions.

As formative research, Dadkhah found that first-year university students (*n* = 216) unanimously liked the idea of a Facebook-delivered healthy eating intervention [51]. Some studies that included social media in their interventions asked their participants how acceptable or useful they found it. Ashton et al. [6] asked participants to rank the usefulness of each component of their complex intervention via a survey. The Facebook component was rated 3.5 to 4.2 (range 1: low-5 high) for each question. This was ranked on the lower end of effectiveness of all the eight intervention components. Face-to-face components were consistently ranked higher. Beetham [40] found a mean ranking of 2.8 (range 1–5) for perceived benefit of a Facebook support group, which was also the lowest score for all 13 strategies used in the intervention. In contrast, Napolitano et al. [39] found that 97% of participants found their Facebook intervention helpful (at least three on a four-point scale) and 100% would recommend the program to others. Dadkhah [51] also reported that 99% of her participants would recommend that the intervention be offered to future university students. These studies with the highest levels of engagement and acceptability used private Facebook groups to provide health information and tips, post events and polls and other engaging and interactive content.

### Aim 5: Understand how young adults use social media for nutrition-related behaviors

The six studies included in the qualitative synthesis used a range of methodologies. One study used multiple methods, but others used focus groups, semi-structured individual interviews, open-ended survey questions and one content analysis of an online forum. Only one study used a ‘lived experience’ approach where they asked participants to describe their usual (day-to-day) experience of using social media. The others studies were either process evaluations of an intervention or were part of formative research; therefore asked open-ended questions specifically relating to an intervention (i.e. not of their usual use of social media).

Several themes emerged from the qualitative explorations of using social media including information dissemination and the “good and bad” social aspects of social media; potential for social support and social undesirability. Table 3 summarizes the themes from the qualitative studies and provides examples of the studies contributing to each theme. The three main themes found were using social media for disseminating information, providing social support (the good) and the potential social undesirability (the bad) of using social media.

**Table 3:** Qualitative study themes

Theme	Study findings	Study design
Information dissemination	First-year university students unanimously liked the idea of posting healthy-eating and physical-activity tips on a Facebook page. Students suggested the posting of nutrition information on menu items, greater food variety with healthier options, less variety for unhealthy food in dining halls, and coordination of university based activities as ways to improve healthy eating and physical activity [51].	Focus groups; formative research (informing trial design)
	In focus group and individual interviews, young adults described the connection between food and social media. It was associated with increased food choices [65].	Focus groups and interviews
	In an online weight-loss support community, the most discussed topics on were: healthy food, clothing, calorie counting g, workouts, looks, habits, support, and unhealthy food [48].	Observational; Retrospective cohort
Social support	Young university students indicated there were a few instances (exceptions) where it MAY be ok to share health information via social media. This may be when you have achieved something “He really liked doing that, to share with people, and he had loads of people “liking” it, and he felt that was quite inspiring.” [52]	Focus groups
	In focus group and individual interviews, young adults reported it was common for people to post food and exercise pictures/updates on social media particularly regarding accomplishments [65].	Focus groups; formative research (informing trial design)
	In an online weight-loss support community, support was a common theme found in the comments (encouraging the people posting the topic) [48].	Observational; Retrospective cohort
Social undesirability	Most participants reported that they felt supported to make lifestyle changes by the study team but less so by others within the group [41].	Process evaluation of a Twitter-delivered intervention.
	Young adults felt that social media features of apps were unnecessary, inappropriate and off-putting due to the social undesirability i.e. it’s slightly embarrassing. “If this popped up, I think people would laugh at me.” [52]	Focus groups
	In a process evaluation where participants were exposed to a Facebook page, some participants “lurked” on Facebook (i.e. they saw posts but did not want to interact with them.) Some did not feel comfortable sharing their personal information on Facebook, particularly because their friends could see it. They also found that participants felt that Facebook was “too public” to post information about being in a weight loss program [46].	Process evaluation of a Facebook-delivered intervention.

#### Disseminating information

Formative, process evaluations and other studies suggested that young adults are interested in using social media for learning about nutrition-related information. Young adults reported that social media channels were a useful platform to learn about new recipes and healthy eating.

#### Social support: the good and bad

Social media can provide social support, inspiration and/or motivation to change or maintain healthy behaviors. This was the intention by researchers in the intervention design for several studies and was identified as important by participants in exploratory studies; however, one study highlighted an important ‘caveat’ for this use. Young university students indicated there were only a few instances where it may be acceptable to share their health information via social media, such as achieving a personal health-related goal as this can solicit positive feedback from social networking friends [52]. The process evaluations were not clear about how effectively the interventions used social media for social support by the participants. Several studies found that there was a social undesirability of using social media for health-related interventions. Young adults were reluctant to post weight-related information on their personal social media accounts. Some participants reported that it was more acceptable to post information about health-related interventions within social media groups where members had a common interest/goal.

## Synthesis

This mixed methods synthesis uses the qualitative findings to help explain the quantitative findings about use and engagement. In interventions where social media was either a major or a minor component of a complex intervention, social media was often not (or infrequently) used by participants. The qualitative studies help to shed light on the potential reasons why people would and would not use the social media component. The majority of studies used social media to disseminate information, and this appears to be an acceptable and desirable use of social media confirmed by the qualitative studies. However, information dissemination may not be interactive as it only describes the dissemination of information, not the reading of or interacting with the information. Engagement metrics may not detect post views or social support activities provided via other forums, as it is difficult to detect when a post has been read or not. Users can read a post without ‘clicking’ on it or sharing it, however, when they do share a post, they are “interacting” with it and allow the interaction to be collected by engagement metrics. There is a potential to use social media for its social networking and support capabilities by inspiring and soliciting positive feedback from social networking friends, however, for health professionals, this must be managed carefully as professionals may not be able to manage organically created social interactions. Formative research indicated that some young adults did not want to use social media for weight, but process evaluations did not evaluate the effectiveness of social media for social support. There appears to be a trade-off between the potential benefits of receiving social support and when the social aspect of social networking around nutrition-related outcomes becomes undesirable. The undesirability may lead to decreased engagement on the social media platform throughout a weight loss intervention. Utilizing a private social media account, as found in Chung et al.’s [41] study using private Twitter accounts, may be an effective solution in addressing this privacy issue, as they reported that the overweight group, which increased their fruit and vegetable intakes and decreased their sugar-sweetened beverage intakes by the end of the trial, tweeted more than the healthy weight group.

## Discussion

Social media is being used as one aspect in complex interventions to provide information to participants and as an avenue to provide social support for behavior change. Young adults appear to find information provision an acceptable and desirable way to use a social media platform, however, using social media as a tool to enable social support is multi-faceted. The majority of interventions included in this review were not effective for improving outcomes such as weight, BMI, or dietary intake when compared with control groups. As most interventions included multiple components, it is difficult to attribute effectiveness of social media (the common component) itself. Effectiveness also may have been influenced by engagement and overall use of social media, or the use of social media by the researchers themselves, or by the other components of the interventions. Outcomes may be influenced by differences in implementing social media, for example a well-designed, visually-attractive Facebook page, highlighting interesting topics and including relevant language used to communicate via social media by the researchers [53] may lead to improved outcomes. However, the information provided by the included studies did not allow for this to be analyzed. Private Facebook pages were used in the studies included in this review with highest and lowest engagement, therefore this is more than the channel itself influencing engagement, and therefore potential outcomes are influenced by many factors out of the control of the study.

We found that engagement with social media in interventions varied widely from 3 to 69%. There were differences between the studies in how they measured engagement (i.e. by likes’ and comments on study posts about recipes/nutrition, number of participant posts, responses to events, page views and how they reported engagement). These metrics reflect the lack of additional tools available and are probably inadequate for truly assessing ‘engagement’ and Neiger [54] argues the metric for engagement should go beyond ‘liking’ to include a level of sharing and co-creating content. Low engagement with social media in interventions, regardless of the measure used, is widely reported as a problem [18, 26]. In several trials in our review, social media was the least used – and lowest ranked for usefulness – component of the interventions. It is unclear whether this differs by age group and whether there are other factors leading to poor engagement, such as the quality of the intervention (i.e. design, engaging content provided) or personal factors, such as interest in the intervention itself or the stage of change of the individual. Social media use differs by age in general [55], and in formative research examining interest in a Twitter-delivered weight loss intervention, Waring et al. [56] found that the youngest group of women included in their study were the least likely to be interested in a Twitter-delivered weight loss intervention; 62% in 21–29 years, 96% in 30–34 years and 77% in 35–45 years.

The qualitative analyses support our understanding of how and why social media was used and whether it may be effective at changing behaviors of nutrition-related outcomes, and provided insights into the meaning behind engagement. Young adults identified that social media was useful to deliver information and encourage learning. A common finding between real-life, lived experience research and intervention planning was that social media provides a valuable tool for sharing information. Researchers often used a social media platform for information provision [23] and as most people are familiar with social media platforms, it prevents people from downloading ‘yet another app’ to use, and for the researchers to have to maintain a website. This use of social media does not appear to be any more or less effective than providing static information on a different website [20] and using social media may be more difficult to determine engagement from standard analytics measures. Most RCTs also included a website as a part of their intervention, and sometimes links on Facebook were directed to the website providing more in-depth information. Researchers when designing future interventions should examine whether this duplication is redundant. The very definition of social media indicates that its purpose is for sharing, collaboration and interactivity. Using it with a top-down approach as an information-delivery tool only, may not use its capabilities to their full potential.

In addition to engagement with information resources, social media has the potential to be used for providing social support. There is a body of evidence recognizing the importance of social support in enabling behavior change [57–59]. Online social networking can provide a social support network for people seeking to change health-related behaviors and provide an easy way of connecting with others who have similar goals and beliefs. Young adults reported that using social media to post about weight-related information was socially undesirable, which may explain why participants did not have high levels of engagement with social media in some of the included studies: they may be unwilling to share details of engagement with weight-related social media interventions with their social support group. There may be different issues using social media for sensitive and potentially stigmatizing subjects such as weight loss and obesity [14, 60] and this should be considered during intervention or campaign design. For example, many of the included studies’ authors stated in the methods that the social media channel was intended for use as social support; however, when examining the process evaluation or engagement metrics, this may not have been used as intended with limited engagement and conversations taking place on the private social media groups set up by the researchers. This confirms findings from other systematic reviews exploring social media for weight loss in all adults: the private discussion forums that were a component of the interventions were not well used by participants [24] indicating that the key may be to tap into existing social networks instead of attempting to create artificial ones. In “real-world” settings, some individuals use social media for social support in order to facilitate behavior change for encouragement and motivation [61], although this use is not ubiquitous. In one survey, 12% of young adults said they used an online support group, blog, or other online community [62]. The group on Reddit, a social media channel, called “LoseIt” is an example of a social support group for weight loss and is described in the reviewed paper by Pappa et al. They demonstrated a huge variation in user engagement as well (i.e. posting and responding in forums) [48], therefore, although engagement may vary, given the differences in “people”, it may still be beneficial to have different types of social media for individuals who benefit from it. Different people use social media differently organically as well as in a clinical trial setting; engagement metrics may not capture social support behaviors that occur in different channels (e.g. a purposeful Reddit group compared with an organically adaptive Facebook social network). Social media user types may be characterized as “super-users” (high levels of engagement, posting, commenting and sharing; these user types were found in several of the included studies), sharers (sharing posts and/or tagging friends in posts), posters (posting their own original content), “lurkers” (viewing information, but not necessarily commenting or sharing, therefore are not picked up by engagement metrics) [46, 61] and those with no engagement (not viewing or engaging with content in any way). Researchers should expect different levels of engagement with social media, and complex interventions need to be cognizant of their audience and need to be designed to account for young adults’ variation in social media use as well as their other components and may need a variety of techniques to reach different groups.

Limitations of this review include that it was difficult to measure the additive benefits of the social media component in complex interventions, as it was only one component of multi-component complex interventions, that could be a small or large component of the intervention, and as social media was used differently between the studies. As previously argued by Vandelanotte [28] and Lim [29], randomised trials, even if they are of complex interventions, may not be the best measures of effectiveness for social media interventions and no ecological studies nor health promotion campaigns were found. It was also difficult to compare engagement metrics between studies as the metrics reported varied, highlighting a gap in tools available to researchers. Many of the qualitative and engagement studies were hypothetical rather than real world or lived experience, or content analyses of actual social media posts, which limits our understanding of how individuals actually use social media. The generalizability of this review should reflect the populations and settings of included studies were from the USA with mainly overweight participants recruited from universities.

## Conclusions

Social media is a widely acceptable media for delivering nutrition-related information to young adults. To date, its use in interventions does not seem to have an additive effect for weight loss or reducing BMI. Although young adults are open to receiving healthy eating and recipe tips through social media, they are reluctant to share personal weight-related information with their online social networks. Future research should consider how to best engage with different types of social media users and how to target their social media more effectively to support, facilitate social and peer-to-peer support for young adults in making healthier choices.

## Additional file

Additional file 1: Supporting information: Search strategy terms, method for classifying social media use, quality appraisals and characteristics of interventions. (PDF 381 kb)

## Abbreviations

BMI: Body mass index
MMAT: Mixed Methods Appraisal Tool
ONS: Online networking site
RCT: Randomized controlled trial
SMS: Short message service
SNS: Social networking site
SSB: Sugar-sweetened beverage
