# Supplementary material for: Social media use for nutrition outcomes in young adults: a mixed-methods systematic review
Source: Int J Behav Nutr Phys Act. 2018 Jul 24;15:70. doi: 10.1186/s12966-018-0696-y (PMC6057054; doi:10.1186/s12966-018-0696-y)
Supplement: Supplementary file 1 — Supporting information: Search strategy terms, method for classifying social media use, quality appraisals and characteristics of interventions. (PDF 381 kb) [file 12966_2018_696_MOESM1_ESM.pdf]

Supporting information

Table S1: Search strategy terms

Keywords included any combination of a social media term AND young adult term AND nutrition term. (("social media" OR "social networking site*" OR "social network* website*" OR "online network*" OR "online social network*" OR "blog" OR facebook OR "web 2.0" OR twitter OR youtube OR snapchat OR instagram OR pinterest OR tumblr OR bebo OR flickr OR vimeo OR vine OR periscope OR twitch OR twitter OR google+ OR "google plus" OR qzone OR "sina weibo" OR myspace OR renren OR twoo OR mymfb OR vkontakte)) AND ("young adult*" OR college student OR university student) AND (diet OR nutrition OR obes* OR weight OR food OR eat*) NOT (patient) NOT (disease) NOT (cancer).

Database	Search terms
Proquest-ERIC	("social media" OR "social networking site*" OR "social network*" OR "online network*" OR "online social network*" OR blog* OR facebook OR "web 2.0" OR twitter OR youtube OR snapchat OR instagram OR pinterest OR tumblr OR "google+" OR "google plus" OR qzone OR "sina weibo" OR myspace OR renren OR twoo OR mymfb OR vkontakte) AND ("young adult*" OR college AND student OR university AND student) AND (diet OR overweight OR obes* OR food)
PubMed	("social media"[All Fields] OR "social networking site*"[All Fields] OR "social network* website*"[All Fields] OR "online network*"[All Fields] OR "online social network*"[All Fields] OR "blog"[All Fields] OR facebook[All Fields] OR "web 2.0"[All Fields] OR twitter[All Fields] OR youtube[All Fields] OR snapchat[All Fields] OR instagram[All Fields] OR pinterest[All Fields] OR tumblr[All Fields] OR bebo[All Fields] OR flickr[All Fields] OR vimeo[All Fields] OR vine[All Fields] OR periscope[All Fields] OR twitch[All Fields] OR twitter[All Fields] OR google[All Fields] OR "google plus"[All Fields] OR "sina weibo"[All Fields] OR myspace[All Fields] OR renren[All Fields] OR twoo[All Fields]) AND ("young adult*"[All Fields] OR college[All Fields] AND ("students"[MeSH Terms] OR "students"[All Fields] OR "student"[All Fields]) OR ("universities"[MeSH Terms] OR "universities"[All Fields] OR "university"[All Fields]) AND ("students"[MeSH Terms] OR "students"[All Fields] OR "student"[All Fields])) AND (("diet"[MeSH Terms] OR "diet"[All Fields]) OR ("exercise"[MeSH Terms] OR "exercise"[All Fields]) OR ("overweight"[MeSH Terms] OR "overweight"[All Fields]) OR (obes[All Fields] OR obes'ian[All Fields] OR obesa[All Fields] OR obesas[All Fields] OR obesbolivaiushchego[All Fields] OR obesbolivaniia[All Fields] OR obeschennosti[All Fields] OR obeschermde[All Fields] OR obescities[All Fields] OR obesco[All Fields] OR obescure[All Fields] OR obese[All Fields] OR obese'[All Fields] OR obese's[All Fields] OR obese16[All Fields] OR obesechennost[All Fields] OR obesechennost'[All Fields] OR obesed[All Fields] OR obesegenic[All Fields] OR obesegirls[All Fields] OR obesego[All Fields] OR obeseht[All Fields] OR obesehyperglycemic[All Fields] OR obeseindividuals[All Fields] OR obesen[All Fields] OR obesenam[All Fields] OR obesend[All Fields] OR obeseness[All Fields] OR obeseni[All Fields] OR obesenim[All Fields] OR obesent[All Fields] OR obeseogenic[All Fields] OR obesepcos[All Fields] OR obesephobia[All Fields] OR obesephobia'[All Fields] OR obesepostmenopausal[All Fields] OR obeser[All Fields] OR obeser'[All Fields] OR obeservation[All Fields] OR obeserve[All Fields] OR obeserved[All Fields] OR obeses[All Fields] OR obeset2dm[All Fields] OR obesetic[All Fields] OR obesewomen[All Fields] OR obesez[All Fields] OR obesfenolivanie[All Fields] OR obesfenolivaniia[All Fields] OR obesftorennogo[All Fields] OR obesftorivanie[All Fields] OR obesi[All Fields] OR obesiay[All Fields] OR obesic[All Fields] OR obesica[All Fields] OR obesidad[All Fields] OR obesidad'[All Fields] OR obesidade[All Fields] OR obesidades[All Fields] OR obesidadesevera[All Fields] OR obesidadtlahuac[All Fields] OR obesidady[All Fields] OR obesidadycirugia[All Fields] OR obeside[All Fields] OR obesidomic[All Fields] OR obesidomica[All Fields] OR obesidomics[All Fields] OR obesification[All Fields] OR obesified[All Fields] OR obesify[All Fields] OR obesifying[All Fields] OR obesigen[All Fields] OR obesigenic[All Fields] OR obesigenic'[All Fields] OR obesigenicity[All

	Fields] OR obesigenicos[All Fields] OR obesin[All Fields] OR obesinymphae[All Fields] OR obesiogenic[All Fields] OR obesisty[All Fields] OR obesit[All Fields] OR obesit'a[All Fields] OR obesit'asban[All Fields] OR obesita[All Fields] OR obesita'[All Fields] OR obesita'e[All Fields] OR obesitaksen[All Fields] OR obesitaksessa[All Fields] OR obesitas[All Fields] OR obesitas'[All Fields] OR obesitasban[All Fields] OR obesitasbehandling[All Fields] OR obesitasenheten[All Fields] OR obesitaskirurgi[All Fields] OR obesitaskliniek[All Fields] OR obesitasopererade[All Fields] OR obesitassyndroom[All Fields] OR obesitasu[All Fields] OR obesitat[All Fields] OR obesitatii[All Fields] OR obesitax[All Fields] OR obesitax'[All Fields] OR obesite[All Fields] OR obesitehyperglycemie[All Fields] OR obesiteit[All Fields] OR obesites[All Fields] OR obesitet[All Fields] OR obesiti[All Fields] OR obesitic[All Fields] OR obesitics[All Fields] OR obesities[All Fields] OR obesitis[All Fields] OR obesitiy[All Fields] OR obesitogenic[All Fields] OR obesitological[All Fields] OR obesitologists[All Fields] OR obesitology[All Fields] OR obesitologyinstitute[All Fields] OR obesitv[All Fields] OR obesity[All Fields] OR obesity'[All Fields] OR obesity's[All Fields] OR obesity,[All Fields] OR obesity1[All Fields] OR obesity101[All Fields] OR obesity2[All Fields] OR obesity2016[All Fields] OR obesity530[All Fields] OR obesitya[All Fields] OR obesityand[All Fields] OR obesityassociated[All Fields] OR obesitychanges[All Fields] OR obesitydepartment[All Fields] OR obesitydevice[All Fields] OR obesitydiseases[All Fields] OR obesityendoscopy[All Fields] OR obesityenhanced[All Fields] OR obesityexecutive[All Fields] OR obesitygene[All Fields] OR obesitygenes[All Fields] OR obesityhealth[All Fields] OR obesityin[All Fields] OR obesityinduced[All Fields] OR obesitylinked[All Fields] OR obesitymax[All Fields] OR obesitymedicineinstitute[All Fields] OR obesitynational[All Fields] OR obesitynetwork[All Fields] OR obesitypreventive[All Fields] OR obesityprone[All Fields] OR obesityrelated[All Fields] OR obesityresearch[All Fields] OR obesitys[All Fields] OR obesitysurgery[All Fields] OR obesitythe[All Fields] OR obesitytoolkit[All Fields] OR obesityuniversity[All Fields] OR obesitywas[All Fields] OR obesityweek[All Fields] OR obesityyale[All Fields] OR obesive[All Fields] OR obesivo[All Fields] OR obesiy[All Fields] OR obesiyy[All Fields] OR obeske[All Fields] OR obeskrivliga[All Fields] OR obeskrovlennogo[All Fields] OR obeskrovlennom[All Fields] OR obeskrovlennykh[All Fields] OR obeskrovlivanie[All Fields] OR obeskrovlivaniem[All Fields] OR obeskrovlivanii[All Fields] OR obeskrovlivaniia[All Fields] OR obeskrylivanie[All Fields] OR obeslaktad[All Fields] OR obeslaktade[All Fields] OR obesminva[All Fields] OR obesnich[All Fields] OR obesnosci[All Fields] OR obeso[All Fields] OR obesoconnus[All Fields] OR obesoechenie[All Fields] OR obesofemoria[All Fields] OR obesogammarus[All Fields] OR obesogen[All Fields] OR obesogen'[All Fields] OR obesogene[All Fields] OR obesogeneity[All Fields] OR obesogenes[All Fields] OR obesogenesis[All Fields] OR obesogenic[All Fields] OR obesogenic'[All Fields] OR obesogenica[All Fields] OR obesogenicity[All Fields] OR obesogenico[All Fields] OR obesogenicos[All Fields] OR obesogenics[All Fields] OR obesogenique[All Fields] OR obesogenity[All Fields] OR obesogeno[All Fields] OR obesogenos[All Fields] OR obesogenous[All Fields] OR obesogens[All Fields] OR obesogens'[All Fields] OR obesogeonic[All Fields] OR obesollivaniia[All Fields] OR obesomartin[All Fields] OR obesometric[All Fields] OR obesometrics[All Fields] OR obesophobia[All Fields] OR obesoprotective[All Fields] OR obesos[All Fields] OR obespe[All Fields] OR obespeccheniia[All Fields] OR obespecehniia[All Fields] OR obespecehnnosti[All Fields] OR obespechaet[All Fields] OR obespechaniia[All Fields] OR obespechenia[All Fields] OR obespechenie[All Fields] OR obespecheniem[All Fields] OR obespechenii[All Fields] OR obespecheniia[All Fields] OR obespecheniiavoisk[All Fields] OR obespecheniie[All Fields] OR obespecheniiia[All Fields] OR obespecheniiu[All Fields] OR obespecheniya[All Fields] OR obespecheniye[All Fields] OR obespechennesti[All Fields] OR obespechennio[All Fields] OR obespechennost[All Fields] OR obespechennost'[All Fields] OR obespechennosti[All Fields] OR obespechennsoti[All Fields] OR obespechenost[All Fields] OR obespechenost'[All Fields] OR obespechenosti[All Fields] OR obespechie[All Fields] OR obespechim[All Fields] OR obespechin[All Fields] OR obespechit[All Fields] OR obespechit'[All Fields]

	OR obespechivaet[All Fields] OR obespechivaetcia[All Fields] OR obespechivaetsya[All Fields] OR obespechivaiuschaia[All Fields] OR obespechivaiushchaia[All Fields] OR obespechivaiushchaiushchaia[All Fields] OR obespechivaiushchee[All Fields] OR obespechivaiushchego[All Fields] OR obespechivaiushchei[All Fields] OR obespechivaiushchem[All Fields] OR obespechivaiushchie[All Fields] OR obespechivaiushchii[All Fields] OR obespechivaiushchikh[All Fields] OR obespechivaiushchikkh[All Fields] OR obespechivaiushchim[All Fields] OR obespechivaiut[All Fields] OR obespechival[All Fields] OR obespechivat[All Fields] OR obespechivat'[All Fields] OR obespechneii[All Fields] OR obespechniia[All Fields] OR obespechnost[All Fields] OR obespechnost'[All Fields] OR obespechnosti[All Fields] OR obespencheniia[All Fields] OR obespepechenie[All Fields] OR obesplechenie[All Fields] OR obesplozhennoi[All Fields] OR obespolleri[All Fields] OR obespschennosti[All Fields] OR obespylennoi[All Fields] OR obespylivaiushchaia[All Fields] OR obespylivanie[All Fields] OR obespylivanii[All Fields] OR obespylivaniia[All Fields] OR obesrve[All Fields] OR obesrved[All Fields] OR obess[All Fields] OR obessakharivanie[All Fields] OR obesser[All Fields] OR obesserivanii[All Fields] OR obesses[All Fields] OR obesshumlivanie[All Fields] OR obession[All Fields] OR obessional[All Fields] OR obessionality[All Fields] OR obessisionality[All Fields] OR obessive[All Fields] OR obessivo[All Fields] OR obessmolennogo[All Fields] OR obessmolennyi[All Fields] OR obessmolennym[All Fields] OR obesso[All Fields] OR obessolennaia[All Fields] OR obessolennoi[All Fields] OR obessolivanieproduktov[All Fields] OR obessolivaniia[All Fields] OR obesssional[All Fields] OR obesssurg[All Fields] OR obessurg[All Fields] OR obessus[All Fields] OR obest[All Fields] OR obesta[All Fields] OR obestard[All Fields] OR obestateric[All Fields] OR obestatin[All Fields] OR obestatin's[All Fields] OR obestatins[All Fields] OR obestatinu[All Fields] OR obestatyny[All Fields] OR obeste[All Fields] OR obestechenie[All Fields] OR obester[All Fields] OR obesterics[All Fields] OR obestertics[All Fields] OR obestetic[All Fields] OR obestetics[All Fields] OR obestetric[All Fields] OR obestetrical[All Fields] OR obestetricia[All Fields] OR obestetrics[All Fields] OR obestetricts[All Fields] OR obestia[All Fields] OR obestic[All Fields] OR obestitics[All Fields] OR obestity[All Fields] OR obestives[All Fields] OR obestiy[All Fields] OR obestiy'[All Fields] OR obestran[All Fields] OR obestrical[All Fields] OR obestricians[All Fields] OR obestrics[All Fields] OR obestritics[All Fields] OR obestsvechivaiushchego[All Fields] OR obestsvechivanie[All Fields] OR obestsvechivanii[All Fields] OR obestsvechivaniia[All Fields] OR obesula[All Fields] OR obesulus[All Fields] OR obesum[All Fields] OR obesumbacterium[All Fields] OR obesus[All Fields] OR obesvarad[All Fields] OR obesvarade[All Fields] OR obesvrezhivaniia[All Fields] OR obesy[All Fields]) OR ("food"[MeSH Terms] OR "food"[All Fields])) NOT (("patients"[MeSH Terms] OR "patients"[All Fields] OR "patient"[All Fields]) OR ("disease"[MeSH Terms] OR "disease"[All Fields]) OR ("neoplasms"[MeSH Terms] OR "neoplasms"[All Fields] OR "cancer"[All Fields]))
Scopus	(TITLE-ABS-KEY ("social media" OR "social networking site*" OR "social network*" OR "online network*" OR "online social network*" OR blog* OR facebook OR "web 2.0" OR twitter OR youtube OR snapchat OR instagram OR pinterest OR tumblr OR "google+" OR "google plus" OR qzone) OR TITLE-ABS-KEY ("sina weibo" OR myspace OR renren OR twoo OR mymfb OR vkontakte) AND TITLE-ABS-KEY ("young adult*" OR college AND student OR university AND student) AND TITLE-ABS-KEY (diet OR exercise OR overweight OR obes* OR food) AND NOT TITLE-ABS-KEY (patient OR disease OR cancer))
Emerald	("social media" OR "social networking site*" OR "social network*" OR "online network*" OR "online social network*" OR blog* OR facebook OR "web 2.0" OR twitter OR youtube OR snapchat OR instagram OR pinterest OR tumblr OR "google+" OR "google plus" OR qzone OR "sina weibo" OR myspace OR renren OR twoo OR mymfb OR vkontakte) AND ("young adult*" OR college AND

	student OR university AND student) AND [Abstract:(diet OR overweight OR obes* OR food)]
ProQuest Central	(all("social media") OR all("social networking site*") OR all("social network* website*") OR all("online network*") OR all("online social network*") OR all(blog*) OR all(facebook) OR all("web 2.0") OR all(youtube) OR all(snapchat) OR all(instagram) OR all(pinterest) OR all(tumblr) OR all(bebo) OR all(flickr) OR all(vimeo) OR all(vine) OR all(periscope) OR all(twitch) OR all(twitter) OR all(google+) OR all("google plus") OR all(qzone) OR all("sina weibo") OR all(myspace) OR all(renren) OR all(twoo) OR all(mymfb) OR all(vkontakte)) AND (all("young adult*")) AND (all(diet) OR all(food) OR all(eat*) OR all(weight) OR all(obes*)) NOT (all(patient) OR all(disease) OR all(cancer))
EBscohost:MEDLINE; CINAHL Plus; Business Source Complete, Communication & Mass Media Complete	(("social media" OR "social networking site*" OR "social network* website*" OR "online network*" OR "online social network*" OR "blog" OR facebook OR "web 2.0" OR twitter OR youtube OR snapchat OR instagram OR pinterest OR tumblr OR bebo OR flickr OR vimeo OR vine OR periscope OR twitch OR twitter OR google+ OR "google plus" OR qzone OR "sina weibo" OR myspace OR renren OR twoo OR mymfb OR vkontakte)) AND ("young adult*" OR college student OR university student) AND (diet OR nutrition OR obes* OR weight OR food OR eat*) NOT (patient) NOT (disease) NOT (cancer)
Web of Science (includes: Science Citation Index Expanded; social sciences;	TOPIC: ("social media" OR "social networking site*" OR "social network*" OR "online network*" OR "online social network*" OR blog* OR facebook OR "web 2.0" OR twitter OR youtube OR snapchat OR instagram OR pinterest OR tumblr OR "google+" OR "google plus" OR qzone OR "sina weibo" OR myspace OR renren OR twoo OR mymfb OR vkontakte) AND TOPIC: (diet OR overweight OR obes* OR food) AND TOPIC: ("young adult*" OR "college student*" OR "university student*") NOT TOPIC: (patient or disease or cancer)
OVID: psycARTICLES full text, JBI, EBM reviews-acp, cochrane, hta, nhs, AMED, psycBOOKS, PsycINFO 1987-2017	((social adj1 media) or (social adj1 network*) or (online adj1 network*) or (online adj1 social) or blog* or facebook or (web adj1 "2.0") or twitter or youtube or snapchat or instagram or pinterest or tumblr or google+ or (google adj1 plus) or qzone or weibo or myspace or renren or twoo or mymfb or vkontakte).mp. [mp=ti, ot, ab, tx, ct, sh, hw, kw, sa, tc, id, tm] and ((young adj1 adult*) or (college adj1 student*) or (university adj1 student*)).mp. [mp=ti, ot, ab, tx, ct, sh, hw, kw, sa, tc, id, tm] and (diet or nutrition or obes* or weight or food or eat*).m_titl.) not cancer.mp. not disease.mp. not patient.mp. not disorder*.mp. [mp=ti, ot, ab, tx, ct, sh, hw, kw, sa, tc, id, tm]

Table S2: Method for classifying social media use

Social media use as part of an intervention or an exposure or phenomenon of interest will be categorised into the following categories, previously identified in defining social media:	community-based input, interaction, content-sharing and collaboration.
Types of social media will be categorized into the following categories, previously identified when defining social media:	websites and applications dedicated to forums, microblogging, social networking, social bookmarking, social curation, and wikis.
Additional categories identified by previous reviews include:	commercial online social network websites (built or commissioned by a commercial enterprise, such as an organisation selling weight loss products), research-built social network websites (built or commissioned by the research team), multi-component interventions delivered in part using pre-existing popular online social network sites (studies falling into this category need further categorisation into open or closed social media) (1); studies that observed health behaviours by performing content

	analysis of SNS, assessing social media use via interview or survey, eliciting reactions to public posts on social media, engaging the community by facilitating communication with health care professionals or creating an online community (open or closed);(2, 3) (Williams 2014 (4) categorised social media use into types used, but other than mentioning social media was mostly used as discussion boards, did not systematically categorise use.
Other potential uses may be:	If additional uses are found, new categories will be created. Additionally, the purpose of the social media use categories can include: using social media channels as closed discussion boards; providing health information (closed or open), to enhance social support.

Table S3: Quality Appraisals

Study Id	Overall Quality	Method/study design*	MMAT-screening1**	MMAT-screening2	Question 1***	Question 2	Question 3	Question 4
Dadkhah 2013 [1] (5)	Moderate	1	low	low	low	unclear	high	high
Dennison 2013 (6)	High	1	low	low	low	low	low	high
Vaterlaus 2015 (7)	High	1	low	low	low	low	low	high
Leak 2014 (8)	Moderate	1	low	low	low	low	high	high
Ashton 2017 (9)	High	2	low	low	low	low	low	
Beetham 2015 (10)	Poor	2	low	low	high	high	high	high
Dadkhah 2013 [3] (5)	Poor	2	low	low	high	high	high	high
Godino 2016 (11)	High	2	low	low	low	unclear	low	low
Gow 2010 (12)	Poor	2	low	low	high	high	high	high
Hebden 2014 (13)	High	2	low	low	low	unclear	low	low
Laska 2016 [2] (14)	High	2	low	low	low	unclear	low	low
Lytle 2017 (15)	High	2	low	low	low	high	low	low
Meng 2017 (16)	Moderate	2	low	unclear	high	low	low	high
Napolitano 2013 (17)	High	2	low	low	high	high	low	low
Partridge 2015 (18)	Moderate	2	low	low	low	unclear	high	low
Chung 2017 (19)	Poor	3	high	low	high	high	high	unclear
Dadkhah 2013 [2] (5)	Moderate	3	low	low	high	low		high
Harvey-Berino 2012 (20)	Poor	3	low	high	high	high	unclear	high
Laska 2016 (21)	Poor	3	low	high	unclear	high	unclear	low
Waring 2016 (22)	Poor	4	low	unclear	high	high	high	unclear

Partridge 2016 (23)	Moderate	5	low	high	high	--	--	--
Pappa 2017 (24)	Poor	5	low	unclear	unclear	high	high	--
Merchant 2014 (25)	Moderate	5	low	low	low	high	high	--

*Study design number 1 corresponds to qualitative studies; 2: RCTs; 3: experimental or cohort studies; 4: cross-sectional studies; 5: mixed methods.

**MMAT-screening question #1-Are there clear qualitative and quantitative research questions or objectives or a clear mixed methods question or objective?

MMAT-screening question #2-Do the collected data allow address the research question/objective e.g. consider whether the follow-up period is long enough for the outcome to occur for longitudinal studies or study components?

***Questions 1-4 differ depending on the study design.

Qualitative studies were assessed using MMAT1.1-1.4:

MMAT-1.1. Are the sources of qualitative data-archives documents informants observations-relevant to address the research question/objective?

MMAT-1.2. Is the process for analysing qualitative data relevant to address the research question/objective?

MMAT-1.3. Is appropriate consideration given to how findings relate to the context, e.g., the setting, in which the data were collected?

MMAT-1.4. Is appropriate consideration given to how findings relate to researchers' influence e.g. through their interactions with participants?

RCTs were assessed using MMAT2.1-2.4:

MMAT-2.1. Is there a clear description of the randomization (or an appropriate sequence generation)?

MMAT-2.2. Is there a clear description of the allocation concealment (or blinding when applicable)?

MMAT-2.3. Are there complete outcome data (80% or above)? for All outcomes

MMAT-2.4. Is there low withdrawal/drop-out (below 20%)?

Experimental or cohort studies were assessed using MMAT3.1-3.4:

MMAT-3.1. Are participants/organizations recruited in a way that minimizes selection bias?

MMAT-3.2. Are measurements appropriate/clear origin or validity known or standard instrument; and absence of contamination between groups when appropriate regarding the exposure/intervention and outcomes? for All outcomes

MMAT-3.3. In the groups being compared-exposed vs. non-exposed with intervention vs. without; cases vs. controls; are the participants comparable or do researchers take into account/control for the difference between these groups?

MMAT-3.4. Are there complete outcome data-80% or above and when applicable an acceptable response rate-60% or above or an acceptable follow-up rate for cohort studies-depending on the duration of follow-up?

Cross-sectional studies were assessed using MMAT4.1-4.4:

MMAT-4.1. Is the sampling strategy relevant to address the quantitative research question-quantitative aspect of the mixed methods question?

MMAT-4.2. Is the sample representative of the population understudy?

MMAT-4.3. Are measurements appropriate-clear origin or validity known or standard instrument?

MMAT-4.4. Is there an acceptable response rate-60% or above?

Mixed methods studies were assessed using MMAT5.1-5.3
MMAT-5.1. Is the mixed methods research design relevant to address the qualitative and quantitative research questions/objectives, or the qualitative and quantitative aspects of the mixed methods question/objective?

MMAT-5.2. Is the integration of qualitative and quantitative data or results relevant to address the research question objective?
MMAT-5.3. Is appropriate consideration given to the limitations associated with this integration e.g. the divergence of qualitative and quantitative data or results?

Table S4: Characteristics of interventions, including social media use

Author, Year	Full intervention description
Ashton, 2017 (9)	• responsive website/'resource library' : relevant info/resources, e.g. fact sheets, support videos (e.g. short cooking videos and demonstration of Gymstick™ exercises) and recommended mobile applications for improving eating habits;<br>• Wearable device for behaviour (e.g. fitness) tracking: A Jawbone™ wearable physical activity tracker with associated mobile phone application (UP app) to assist in goal setting and self-monitoring of key health behaviours;<br>• In-person (face-to-face) group sessions: In-person (face-to-face) group sessions;<br>• Personalised food and nutrient report: comparing intakes to Australian food and nutrient recommendations;<br>• Private social media group: a private Facebook discussion group to facilitate social support, send reminders for upcoming face-to- face sessions and send notifications for new material added to the website;<br>• A Gymstick™ resistance band, for home-based strength training with linked routines available on the website.
Beetham, 2015 (10)	• Responsive website/'resource library' : Stoplight Diet information was given via FB group;<br>• Wearable device for behaviour (e.g. fitness) tracking: pedometers;<br>• In-person (face-to-face) group sessions.: 8 weekly sessions for 90 minutes. led by clinical psychology masters students;<br>• Private social media group:: expected to post once weekly in their respective Facebook group during the 8-week intervention. Each Facebook group consisted only of members from the subgroups (n = 7-9), as well as the lifestyle coaches from their groups. The privacy settings were established in such a way that no other individuals were able to access the groups or read what was posted in them. The facilitators used these groups to share additional tips and prompt feedback about the weekly lesson. Each group received the same information on the same day, selected from the categories: nutrition, physical activity, inspirational quotes, reminders to read weekly material and attend sessions, and self-compassion;<br>• Other behaviour tracking/monitoring: The self-monitoring behaviours were recorded on a Google Doc that was accessible from web-enabled devices or smartphones;<br>• Telephone individualised counselling or coaching: weekly phone calls from facilitators.

Chung, 2017 (19)	• Responsive website/'resource library' : Individual and group challenges delivered possibly via website or Twitter<br>• Wearable device for behaviour (e.g. fitness) tracking:: All participants received Fitbit Zip wearable devices and had private Fitbit accounts (measured steps, physical activity intensity and duration and caloric expenditure). There was also an associated app where participants could log their food/diet and see their data in real time.<br>• Personalised food and nutrient report:: All participants received personalized feedback during the second month, which included personalized daily step goals based on their physical activity patterns during the previous month<br>• Private social media group: private Twitter and Fitbit accounts: All participants received Twitter messages (Tweets) from the study team that focused on increasing physical activity, increasing fruit/vegetable intake, and decreasing sugar-sweetened beverage (SSB) intake. Messages centred on making small, incremental changes such that lifestyle modifications were achievable. Participants also received photo-based Twitter messages that were pictures of healthy food options, infographics (graphic representations of information), and website links related to healthy lifestyle tips on the aforementioned areas of focus. We encouraged and reminded participants to wear their Fitbits and log their dietary intake via Tweets that were specifically timed before classes started, meals, and late evening. Participants were encouraged to post questions to the study team or to their Twitter group. We did not require participants to Tweet a specific number of times/day, but the study team posted questions to the group on topics such as "What small change are you going to make this week?" or "What is your biggest struggle this week?" to encourage interactivity and to tailor message content to participant needs. Fitbit accounts were set up to auto tweet daily steps and distance travelled to the assigned private Twitter group so that individuals could see how others were doing, which was the basis of some of the competitions.
Dadkhah, 2013 [3] (5)	• Personalised food and nutrient report: Feedback was provided to participants through an e-mail that contained information about their personal BMI, waist circumference, and number of steps. The e-mail also included details about healthy ranges and recommendation for those measurements<br>• Private social media group: The current study focused on developing and implementing an intensive, 5-days-a-week, Facebook intervention. The basic concepts of the AOM tools were retained; however, the materials were modified to address the unique attitudes and behaviours often associated with first-year college students' weight gain. Factors in the college environment that have been shown to contribute to student weight gain were considered and added to the intervention (e.g., access to all-you-can-eat foods, snacking, and inactivity).Each week for a total of 28 weeks, relevant tools, news articles, educational Web sites, pages, images, or short texts that supported daily tips were posted on the status portion of the Facebook page.<br>• SMS or email messages: Pt's received feedback via email with information about their personal BMI, waist circumference, and no. of steps. Also had info about healthy ranges and recommendations for measurements.

Godino, 2016 (11) and Merchant, 2014 (25)	• Responsive website/ 'resource library': Used to host knowledge-based blog posts on weight, physical activity, diet, and participant success stories. Also contained "Frequently Asked Questions" with information on how to contact the health coach for support. Participants asked to visit website weekly<br>• Telephone individualised counselling or coaching: Participants could speak with the health coach up to ten times as needed via instant messenger, telephone call, or video call for no longer than 15 min. The health coach contacted participants directly if they gained 5 pounds (2·27 kg) or more since their baseline measurement or stopped using apps for more than month<br>• Public social media: Used the social networking features of Facebook to connect participants and allow for social support, accountability, and healthy social norms from existing social networks. Delivered 17 challenges and campaigns that were often culturally themed and promoted changes to weight-related behaviours on at least a monthly basis<br>• Other apps (health or fitness-related): Goal Getter-Used to set weight-related goals and review progress ad hoc. Information could be shared with others. AND Be Healthy-Used to deliver two weight-related challenges per day. Information could be shared with others AND Trend Setter-Used to self-monitor weight, physical activity, and diet daily. Graphs of trends over time could be viewed and shared with others<br>• SMS or email messages: SMS-Used to deliver reminders, facts, and feedback on self-monitored weight, physical activity, and diet on at least a weekly basis. Participants could set message frequency and timing AND email: Used to summarise use of the apps, promote reading of blog posts on the website, and provide reminders of ongoing challenges and campaigns on a weekly basis

Gow, 2010 (12)	Intervention #1<br>• Responsive website/'resource library' : Internet only group received weekly online sessions via Blackboard. Covered topics such as significance of overweight and obesity, the role of the "toxic" college environment, nutrition, increasing physical activity, decreasing sedentary behaviour, mindfulness of hunger and satiety cues, healthy body image, media literacy, and motivation<br>• Private social media group: Discussion board (group discussions) weekly and asynchronous discussion groups facilitated by researcher.<br>• Other behaviour tracking/monitoring: Self-assessments incorporated into Blackboard<br>• Online group sessions: weekly online sessions lasting 45 minutes each, focusing on environmental, personal and behavioural factors. facilitated by trained clinician.<br>Intervention #2<br>• Personalised food and nutrient report: Participants weighed themselves in university gym and reported their weight to principal investigator on Blackboard. Received a graph each week showing individual weight changes via email.<br>• SMS or email messages: Feedback participants received their weekly weight report via email<br>Intervention #3<br>• Responsive website/'resource library' : Combined feedback and internet group received weekly online session via Blackboard. Covered topics such as significance of overweight and obesity, the role of the "toxic" college environment, nutrition, increasing physical activity, decreasing sedentary behaviour, mindfulness of hunger and satiety cues, healthy body image, media literacy, and motivation<br>• Personalised food and nutrient report: Participants weighed themselves in university gym and reported their weight to principal investigator on Blackboard. Received a graph each week showing individual weight changes via email<br>• Private social media group: Discussion board (group discussions) weekly and asynchronous discussion groups facilitated by researcher.<br>• Other behaviour tracking/monitoring: Self-assessments incorporated into Blackboard<br>• SMS or email messages: Combined feedback and internet participants received their weekly weight report via email<br>• Online group sessions: weekly online sessions lasting 45 minutes each, focusing on environmental, personal and behavioural factors.

Harvey-Berino, 2012 (20)	• Responsive website/'resource library' : Students had access to an extensive array of Web-based resources, including a diet and exercise journal, nutrition and exercise educational resources, a bulletin board for group communication, weekly tips, recipes, and a body mass index (BMI) calculator<br>• Personalised food and nutrient report: Students were instructed to record their dietary intake, minutes of physical activity, and weight on a daily basis in an online journal and submit journals each week. The interventionist reviewed all of the diaries weekly and provided feedback to reinforce or shape new behaviours and to identify high-risk situations for problem solving.<br>• Private social media group: 1-hour weekly ''group meetings'' in an online synchronous chat led by an interventionist trained in behaviour modification and online facilitation through the completion of a 45-hour training course. a bulletin board for group communication,<br>• Online group counselling sessions: Students in the class attended 1-hour weekly ''group meetings'' in an online synchronous chat led by an interventionist trained in behaviour modification and online facilitation. Online groups were closed.
Hebden, 2014 (13)	• Personalised food and nutrient report: When participants entered behavioural data into apps, they received tailored motivational advice and feedback regarding population health guidelines.<br>• Private social media group: Internet forums. Participants elected a username and password to access the appropriate Internet forums. Within forums, both participants and investigator LH were able to contribute comments, questions and information. Separate forums were provided for males and females, and also for each behaviour. New information was posted by the investigator LH biweekly (e.g. ideas for quick and easy low-cost meals including vegetarian options).<br>• A Gymstick™ resistance band, for home-based strength training with linked routines available on the website:<br>• In-person individual counselling or coaching: At their baseline appointment, investigator LH (an accredited dietitian)provided (and described the contents of) a ten page printed booklet to all participants. This booklet included the national physical activity guidelines for Australian adults; a recommendation of 45–60 min of moderate intensity physical activity daily for weight management; and a low glycaemic index, higher protein diet (25% of energy as protein), which has been demonstrated to assist with weight management in adults. Information on the number of servings from each of the core food groups, required to achieve this macronutrient distribution (i.e. 25% protein, 45% carbohydrate, 30%fat), within 6500–7000 kJ day for females and 7500–8000 kJ day for males, was provided with example meal plans. During this appointment with the dietitian, all participants were also able to ask questions related to their diet or physical activity. At their baseline appointment, participants selected two of these behaviours to work on during the programme, under the guidance of investigator LH.<br>• Other apps (health or fitness-related): Pt's received access to one smartphone app related to each of the two behaviours they selected. Enabled users to record their behaviours.<br>• SMS or email messages: Pt's received gender-specific SMS text messages and 2 emails about two behaviours (physical activity and sedentary behaviours, fruit and veg intake, energy dense take away and SSB) every week for 12-weeks.

Laska, 2016 (14) and Lytle, 2017 (15)	• Responsive website/'resource library' : information-providing and tips<br>• In-person (face-to-face) group sessions: could opt to do online, hybrid or in-person classes<br>• Private social media group: the website also included a discussion forum for students to engage with each other on a variety of topics, an "Ask the Expert" section where students could ask confidential questions about a personal challenge or health issue, and a hot topics page where news articles were posted<br>• Other behaviour tracking/monitoring: weight and goal-monitoring<br>• Online group education: Intervention participants could take part in the online version of the CHOICES one-credit course focusing on diet, physical activity, stress management and sleep.
Meng, 2017 (16)	Intervention all arms:<br>• Private social media group: healthy eating community created on ning.com; each group page consisted of 3 modules: 1 group goal; 2) self-track message wall to post their F consumptions 3)bar graph illustrating weekly summaries<br>• Other behaviour tracking/monitoring: Reported self-tracking of their fruit and veg consumption three times a week with group members present. Page had graphs of members consumption and a message wall where people could leave comments<br>control<br>• Responsive website/'resource library' : Accessed the web page and reported self-tracking of fruit and veg consumption without group members present<br>• Other behaviour tracking/monitoring: Reported self-tracking of their fruit and veg consumption three times a week without group members present

Napolitano, 2013 (17)	Intervention #1<br>• Private social media group: Private FB group. Intervention content such as handouts and podcasts. Access to polls and healthy activity or eating event invitations. Used group postings and messages. Suggested calorie intake and physical activity levels.<br>Intervention #2<br>• Personalised food and nutrient report: Received personalised feedback via weekly summary reports and had a buddy to act as a support person.<br>• Private social media group: Private FB group. Intervention content such as handouts and podcasts. Access to polls and healthy activity or eating event invitations. Used group postings and messages. Suggested calorie intake and physical activity levels.<br>• Other behaviour tracking/monitoring: Facebook Plus participants: (i) set weight loss goals with a study staff member, which were adjusted as necessary to a maximum of 2 pounds per week; (ii) received tips on effective self-monitoring of food and physical activity; (iii) identified a non-study affiliated ‘‘buddy’’ who received an online assent directly from the participant to agree to be an identified support; and (iv) received a digital scale, pedometer, Calorie King book, measuring utensils.<br>• SMS or email messages: The text messages were designed to encourage, reinforce, and provide brief feedback regarding self-monitoring of calorie, physical activity, and weight goals. Messages were programmed at random intervals, with a different type of message being sent each day so that the participant would not become habituated to the type of message sent and response required. There were three types; prompt for self-monitoring data, immediate tailored feedback from monitoring reports and tips based on self-identified high risk behaviours.
Pappa, 2017 (24)	Public social media: Reddit is a public social media site. /r "LoseIt" is a subreddit community where people interact about weight loss issues.

Partridge, 2015 (18) and Partridge, 2016 (23)	• Responsive website/ ‘resource library’: Access to resources on a study website. Included resources on healthy eating, meal ideas, physical activity planner, staying healthy in the holidays<br>• Private social media group: community blog<br>• Telephone individualised counselling or coaching: 5 personalized coaching calls from accredited practicing dietitians; helped participants set goals, discussed barriers/enablers and their progress. Calls b/n 10-15 minutes<br>• Other behaviour tracking/monitoring: Self-monitoring on mobile phone apps<br>• Other apps (health or fitness-related): Access to resources on password protected mobile phone apps that provided education and allowed for self-monitoring<br>• SMS or email messages: 8 motivational text messages weekly focusing on four behaviours including SSB, fruits and vegetables, physical activity and food prepared away from home/takeout, 1 email weekly (reiterating messages from the texts and provided links to mobile phone apps)<br>• paper: Mailed an 18 page booklet summarizing the Australian National Dietary and Physical Activity Guidelines, sample meal plans, examples of serving sizes<br>control<br>• Responsive website/‘resource library’ : Access to a different website that contained the 2 page information handout, consent form, study information and contact information<br>• Telephone individualised counselling or coaching: Introductory call at week 0 (but no coaching given)<br>• SMS or email messages: 4 text messages that restated information from the handout<br>• paper: Printed 2-page handout with dietary and physical activity guidelines

References

1. Maher CA, Lewis LK, Ferrar K, Marshall S, De Bourdeaudhuij I, Vandelanotte C. Are health behavior change interventions that use online social networks effective? A systematic review. J Med Internet Res. 2014;16(2):e40.
2. Yonker LM, Zan S, Scirica CV, Jethwani K, Kinane TB. “Friending” Teens: Systematic Review of Social Media in Adolescent and Young Adult Health Care. J Med Internet Res. 2015;17(1):e4.
3. Moorhead SA, Hazlett DE, Harrison L, Carroll JK, Irwin A, Hoving C. A new dimension of health care: systematic review of the uses, benefits, and limitations of social media for health communication. Journal of medical Internet research. 2013;15(4).
4. Williams G, Hamm MP, Shulhan J, Vandermeer B, Hartling L. Social media interventions for diet and exercise behaviours: a systematic review and meta-analysis of randomised controlled trials. BMJ open. 2014;4(2):e003926.
5. Dadkhah M. DEVELOPMENT AND EVALUATION OF THE AMERICA ON THE MOVE: Colorado State University; 2013.
6. Dennison L, Morrison L, Conway G, Yardley L. Opportunities and Challenges for Smartphone Applications in Supporting Health Behavior change: Qualitative Study. J Med Internet Res. 2013;15(4):e86-e.
7. Vaterlaus JM, Patten EV, Roche C, Young JA. #Gettinghealthy: The perceived influence of social media on young adult health behaviors. Comp Human Behav. 2015;45:151-7.
8. Leak TM, Benavente L, Goodell LS, Lassiter A, Jones L, Bowen S. EFNEP Graduates' Perspectives on Social Media to Supplement Nutrition Education: Focus Group Findings From Active Users. Journal of Nutrition Education & Behavior. 2014;46(3):203-8.
9. Ashton LM, Morgan PJ, Hutchesson MJ, Rollo ME, Collins CE. Feasibility and preliminary efficacy of the 'HEYMAN' healthy lifestyle program for young men: a pilot randomised controlled trial. Nutr J. 2017;16(1):2.
10. Beetham R. In-person vs. online small changes weight loss program for undergraduate females. Ann Arbor: Northern Arizona University; 2015.
11. Godino JG, Merchant G, Norman GJ, Donohue MC, Marshall SJ, Fowler JH, et al. Using social and mobile tools for weight loss in overweight and obese young adults (Project SMART): a 2 year, parallel-group, randomised, controlled trial. The Lancet Diabetes & Endocrinology. 2016;4(9):747-55.
12. Gow RW, Trace SE, Mazzeo SE. Preventing weight gain in first year college students: an online intervention to prevent the "freshman fifteen". Eating behaviors. 2010;11(1):33-9.
13. Hebden L, Cook A, van der Ploeg HP, King L, Bauman A, Allman-Farinelli M. A mobile health intervention for weight management among young adults: a pilot randomised controlled trial. J Hum Nutr Diet. 2014;27(4):322-32.
14. Laska MN, Lytle LA, Nanney MS, Moe SG, Linde JA, Hannan PJ. Results of a 2-year randomized, controlled obesity prevention trial: Effects on diet, activity and sleep behaviors in an at-risk young adult population. Preventive medicine. 2016;89:230-6.
15. Lytle LA, Laska MN, Linde JA, Moe SG, Nanney MS, Hannan PJ, et al. Weight-Gain Reduction Among 2-Year College Students: the CHOICES RCT. Am J Prev Med. 2017;52(2):183-91.
16. Meng JB, Peng W, Shin SY, Chung M. Online Self-Tracking Groups to Increase Fruit and Vegetable Intake: A Small-Scale Study on Mechanisms of Group Effect on Behavior Change. J Med Internet Res. 2017;19(3):15.
17. Napolitano MA, Hayes S, Bennett GG, Ives AK, Foster GD. Using Facebook and text messaging to deliver a weight loss program to college students. Obesity. 2013;21(1):25-31.
18. Partridge SR, McGeechan K, Hebden L, Balestracci K, Wong AT, Denney-Wilson E, et al. Effectiveness of a mHealth Lifestyle Program With Telephone Support (TXT2BFiT) to Prevent Unhealthy Weight Gain in Young Adults: Randomized Controlled Trial. JMIR mHealth and uHealth. 2015;3(2):e66.
19. Chung AE, Skinner AC, Hasty SE, Perrin EM. Tweeting to Health: A Novel mHealth Intervention Using Fitbits and Twitter to Foster Healthy Lifestyles. Clin Pediatr. 2017;56(1):26-32.
20. Harvey-Berino J, Pope L, Gold BC, Leonard H, Belliveau C. Undergrad and overweight: an online behavioral weight management program for college students. Journal of nutrition education and behavior. 2012;44(6):604-8.

21. Laska MN, Sevcik SM, Moe SG, Petrich CA, Nanney MS, Linde JA, et al. A 2-year young adult obesity prevention trial in the US: Process evaluation results. Health promotion international. 2016;31(4):793-800.
22. Waring ME, Schneider KL, Appelhans BM, Simas TA, Xiao RS, Whited MC, et al. Interest in a Twitter-delivered weight loss program among women of childbearing age. Transl Behav Med. 2016;6(2):277-84.
23. Partridge SR, Allman-Farinelli M, McGeechan K, Balestracci K, Wong AT, Hebden L, et al. Process evaluation of TXT2BFiT: a multi-component mHealth randomised controlled trial to prevent weight gain in young adults. The international journal of behavioral nutrition and physical activity. 2016;13:7.
24. Pappa GL, Cunha TO, Bicalho PV, Ribeiro A, Couto Silva AP, Meira W, Jr., et al. Factors Associated With Weight Change in Online Weight Management Communities: A Case Study in the LoseIt Reddit Community. J Med Internet Res. 2017;19(1):e17.
25. Merchant G, Weibel N, Patrick K, Fowler JH, Norman GJ, Gupta A, et al. Click "like" to change your behavior: A mixed methods study of college students' exposure to and engagement with Facebook content designed for weight loss. J Med Internet Res. 2014;16(6):182-201.
